# Letter from Dr. Newman

**Published:** 1867-08

**Authors:** Robert Newman

**Affiliations:** 118 W. Houston Street, New York


The following, by Dr. R. Newman, one of the Committee appointed by the "Medical Society of the State of New york," we publish with pleasure, hoping that such of our readers as have any information on the subject of "consanguineous marriages," will communicate it by letter to us, or directly to Dr. Newman of New York.

We feel an earnest desire to promote, as far as in our power, an extensive statistical report on this subject.

118 W. Houston Street,
*New York*, July, 1867.

Sir,—

At the late meeting of the "Medical Society of the State of New York," it was resolved: "That a Committee be appointed to investigate and report upon the result of consanguineous marriages, &c."

If such marriages come under your observation, you will confer a favor by answering the following questions, and transmitting such report, before November next, to the undersigned, one of the Committee appointed:

1. Name (initials) and age of HUSBAND.
2. Nativity.
3. Age when married.
4. Constitution.
5. Health, deformities, peculiar diathesis.
6. Health of his family, hereditary diseases, deformities, &c.

---

7. Name (initials) and age of WIFE.
8. Nativity.
9. Age when married.
10. Constitution.
11. Health, deformities, peculiar diathesis.
12. Health of her family, hereditary diseases, deformities, &c.

---

13. How are the parties related to each other?
14. How long married?
15. How many children, or sterility?
16. Abortions; cause; how many, and at what period?
17. Children died, at what ages and from what diseases?
18. The constitution, age and present health of living children, deformities, mental conditions, idiocy, cretinism, deaf, mute, blind, epilepsy, albinism, insane, &c.
19. Remarks and other information.

Hoping to receive your co-operation for the advancement of medical science,

I remain yours, most respectfully,

ROBERT NEWMAN, M. D.

